# Retraction: The PI3K/AKT/mTOR Signaling Pathway Is Overactivated in Primary Aldosteronism

**DOI:** 10.1371/journal.pone.0335779

**Published:** 2025-11-03

**Authors:** The PLOS One Editors

After this article [1] was published, concerns were raised regarding results presented in Figs 1–2.

Specifically:

• In Fig 1, the APA p-mTOR and IHA p-S6 panels appear similar

• The Fig 2A panel does not present unphosphorylated controls for AKT and S6.

First author HS stated that in Fig 1, the APA p-mTOR panel is correct and the IHA p-S6 panel is incorrect, and provided the original underlying microscopy images. Upon editorial review, multiple additional panels were identified that appear to overlap despite being presented as underlying data for tissue samples from different patients, raising additional concerns about the reliability of the data set and the overall data handling of these results.

Regarding Fig 2, authors HS, YG, and FS stated the levels of AKT and S6 were measured by western blotting in preliminary experiments, and provided cropped blot data for AKT and S6. They stated that AKT and S6 were detected on different blots from their phosphorylated counterparts and author YG stated beta-actin was therefore chosen as a loading control for the phosphorylated proteins. PLOS remains concerned about the validity and reliability of the associated quantified results presented in Figs 2B and 2C.

In light of the above unresolved concerns, the *PLOS One* Editors retract this article.

HS and YG did not agree with the retraction and stand by the article’s findings. FS responded but expressed neither agreement nor disagreement with the editorial decision. FL, QW, BH, XJ, and GN either did not respond directly or could not be reached.

In addition to the concerns above, the *PLOS One* Editors noted that the title of Table 3 describes characteristics of primary aldosteronism patients, but the number of patients included in this table exceeds the number of primary aldosteronism patients described in the article. First author HS stated that Table 3 includes the clinicopathological parameters of both the 45 patients with primary hyperaldosteronism and the 12 normal patients, and they provided the underlying categorized data for each patient in Table 3. The *PLOS One* Editors identified concerns for the Table 3 underlying data, however due to the above unresolved image concerns, PLOS did not investigate the Table 3 concerns further.
